# Identification and comparative analysis of novel major histocompatibility complex-B haplotypes in Indonesian native chickens

**DOI:** 10.5713/ab.250842

**Published:** 2026-03-11

**Authors:** Roshani Fernando, Trisha Nicole Agulto, Prabuddha Manjula, Minjun Kim, Eunjin Cho, Jaewon Kim, Fatmawati Mustofa, Dyah Maharani, Jun Heon Lee

**Affiliations:** 1 Department of Animal Science, Chungnam National University, Daejeon, Korea; 2 Department of Animal Science, University of Peradeniya, Peradeniya, Sri Lanka; 3 Department of Animal Science, Uva Wellassa University, Badulla, Sri Lanka; 4 Department of Bio-AI Convergence, Chungnam National University, Daejeon, Korea; 5 Department of Animal Science, Universitas Diponegoro, Semarang, Indonesia; 6 Department of Animal Breeding and Reproduction, Universitas Gadjah Mada, Yogyakarta, Indonesia

**Keywords:** Indonesian Native Chickens, Kompetitive Allele Specific Polymerase Chain Reaction, Major Histocompatibility Complex-B, Major Histocompatibility Complex-B Haplotypes, Major Histocompatibility Complex-B Single-Nucleotide Polymorphism Panel

## Abstract

**Objective:**

The major histocompatibility complex (MHC) is central to immunological protection. This study represents the assessment of MHC-B haplotype diversity in Indonesian native chickens.

**Methods:**

Six Indonesian native chicken populations were selected as study populations: Merawang (MRG), Pelung (PLG), Black Kedu (KJM), Sentul (STL), Nunukan (NNK), and Gaga (GAG), with 24, 17, 30, 16, 14, and 20 birds from each population, respectively. Samples were genotyped using the MHC-B single-nucleotide polymorphism (SNP) panel. To explore haplotype diversity, the results were analyzed using PHASE 2.1 software.

**Results:**

Genotyping of six Indonesian native chicken populations revealed high haplotype diversity, with a total of 126 distinct haplotypes: 38, 25, 19, 21, 11, and 12 from the MRG, PLG, KJM, STL, NNK, and GAG populations, respectively. Three haplotypes have been identified to be shared: BSNP-IND14 (KJM and STL populations), BSNP-IND26 (KJM, NNK, and MRG populations), and BSNP-IND31 (KJM and MRG populations). Accordingly, all haplotypes obtained from the GAG and PLG populations were unique. Phylogenetic analysis of the results did not reveal a distinct pattern for any population; nonetheless, three subclades were identified, with all six populations represented in each clade. A comparison of MHC-B haplotypes in Indonesian native chickens with MHC-B standard haplotypes shows no distinct clades; however, three possible subclades were also identified. Nevertheless, no Indonesian MHC-B haplotypes matched 100% with the known standard haplotypes, indicating that all Indonesian haplotypes identified in this study are novel. Furthermore, the comparison of Indonesian MHC-B haplotypes to those from other Asian regions reveals that the majority of Indonesian haplotypes cluster with those from Bangladesh, suggesting a shared evolutionary background among South and Southeast Asian chickens.

**Conclusion:**

This study identified unique MHC-B haplotypes in Indonesian native chickens, suggesting that the observed populations are diverse in the MHC-B region and may have variation in immune responses.

## INTRODUCTION

Indonesia is a large tropical island nation in Southeast Asia, providing a habitat for numerous wildlife species and domesticated animals, including poultry. In 2022, Indonesia’s poultry population comprised approximately 3.11 billion broiler chickens, 0.37 billion laying hens, 0.30 billion native chickens, and 56 million ducks, including Muscovy ducks [1]. More than 31 Indonesian indigenous chicken breeds have been identified [2]. Indonesia is a natural habitat for the Red Jungle Fowl (*Gallus gallus*) and shows a wide range of genetic diversity within its wild and native chickens. This diversity highlights Indonesia’s importance in the history and domestication of poultry in Southeast Asia. Genetic studies have identified Indonesia as a major contributor to the regional matrilineal phylogeny, making it a valuable focus for research on biodiversity and conservation of avian genetic resources [3–5]. Chickens are primarily maintained by rural communities, providing a low-cost source of animal protein and meeting economic needs. Moreover, local Indonesian chicken breeds possess distinctive adaptive traits that enable them to survive in harsh climatic, nutritional, and management conditions and develop resistance to diseases in low-input and output systems [6].

Given the native chickens’ high tolerance to various diseases, examining the immune-related genes at the molecular level can provide insights into their genetic diversity. In this context, current immune-related studies highlight an interesting approach on investigating the major histocompatibility complex (MHC). The MHC in chickens, located on chromosome 16, is a crucial gene cluster where genetic diversity is maintained by balancing selection [7–9]. Initial study of the B blood group of the chicken MHC (MHC-B) used serotyping, which revealed links between specific blood groups and their resistance or vulnerability to diseases. Variation in the MHC-B region is well-recognized for its role in conferring resistance to a range of highly pathogenic viral and bacterial diseases, as well as to internal and external parasites in poultry [8,10–14]. Therefore, identifying the MHC-B haplotype in both indigenous and commercial chicken breeds is essential in enhancing management practices and improving productivity in the poultry industry. Initially, a high-density single-nucleotide polymorphism (SNP) panel was developed by Chazara et al [15] to identify the MHC-B diversity. Later, the panel was modified to 101 SNPs (currently used as the 90-SNP panel) by Fulton et al [16] across different chicken lines as a time and cost-effective tool in identifying the genetic diversity of the MHC-B region along with the LEI0258 microsatellite marker [17,18]. Aforementioned, 90-SNP panel (MHC-B SNP panel), which uses the Kompetitive Allele Specific polymerase chain reaction (KASP) chemistry, was successfully used to identify not only the haplotype diversity but also recombination rate and hotspots [16,19,20].

Although Indonesian native chickens are recognized for their genetic diversity and local adaptation [21], the diversity of the MHC B haplotypes remains largely unknown. To date, no study has resolved Indonesian chicken populations into defined MHC-B haplotypes using a high-resolution MHC-B SNP panel approach. As a result, it is unclear whether Indonesian chickens harbor previously characterized MHC-B haplotypes common in other Asian [17,18,22,23] and commercial populations [16,19,20], or whether they possess unique, population-specific haplotypes shaped by local evolutionary pressures.

Therefore, this study aims to characterize the MHC-B diversity of Indonesian indigenous chickens namely: Merawang (MRG), Pelung (PLG), Black Kedu (KJM), Sentul (STL), Nunukan (NNK), and Gaga (GAG), providing a foundation for future research on its role in disease resistance and adaptive variation. Furthermore, the comparison of the MHC-B haplotype diversity among four diverse Asian chicken populations (Sri Lanka, Vietnam, Bangladesh, South Korea) and standard MHC-B haplotypes provide an opportunity to understand the global MHC-B diversity of chicken breeds reared under varying environmental conditions and management practices.

## MATERIALS AND METHODS

### Sample collection and DNA isolation

**Figure 1:**
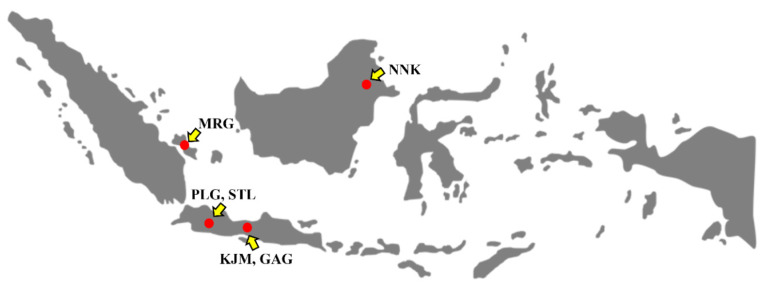
A map showing the sampling locations of Indonesian native chicken populations used in this study: Merawang (MRG) from Bangka Belitung Island; Pelung (PLG) from Cianjur District, West Java Province; Black Kedu (KJM) from Temanggung District, Central Java Province; Sentul (STL) from Majalengka District, West Java Province; Nunukan (NNK) from Nunukan Island, North Kalimantan; Gaga (GAG) from Bantul District and Yogyakarta Kendal District.

A total of 121 samples were collected from 6 Indonesian native chicken breeds randomly, namely MRG (n= 24), PLG (n= 17), KJM (n= 30), STL (n= 16), NNK (n= 14), and GAG (n= 20) from different regions, as shown in Figure 1. The sampling locations and the characteristics of the Indonesian chicken populations used in the current study were previously described [24]. The unequal and relatively small sample sizes among populations reflect practical limitations related to DNA availability and quality from archived samples, rather than intentional sampling bias. Similar sample sizes have been commonly used in previous MHC-B SNP panel studies [22,23] and are sufficient for robust haplotype detection and diversity analyses, given the high resolution of the SNP panel. DNA samples used in this study were previously obtained from the chicken blood collected and preserved as described by Mustofa et al [24]. Briefly, blood samples were obtained from the wing vein, preserved in EDTA tubes at −20°C, and genomic DNA was isolated using the DNA extraction kit (gSYNCTMDNA Extraction Kit, #GS004, GS100, GS300; Geneaid Biotech). The DNA quality and quantity were verified using agarose gel electrophoresis and NanoDrop spectrophotometer (Thermo Fisher Scientific).

### Major histocompatibility complex-B genotyping

A set of 90 SNPs in the high-density MHC-B SNP panel, ranging from SNP marker MHCJ06 to MHC178, was utilized as described by Fulton et al [16,19]. Subsequently, these 90 SNPs were analyzed using a fluorescence-based (FAM and HEX) genotyping method known as KASP. Genotyping quality control of the MHC-B SNP panel included the use of positive controls and negative controls. All SNP assays produced clear and well-separated genotype clusters, with no ambiguous genotype calls detected; therefore, no SNPs or samples were excluded from downstream analyses. However, the MHC138 SNP showed ambiguous genotyping results in a subset of samples. To resolve this issue, locus-specific primers were designed for the MHC138 locus (MHC138F: GGTGTC ACCAGGGAGAGGTTG; MHC138R: CTCCCTCAGGTT GTGAAGCGAAAG), and direct sequencing was performed to accurately determine the corresponding alleles.

### Haplotype identification and nomenclature

Haplotypes were identified using both manual and computational methods. The manual approach followed published procedures [16,19,20]. For the computational process, PHASE haplotype reconstruction was performed using PHASE v2.1. Analyses were run with a burn-in of 1,000 iterations followed by 1,000 sampling iterations with a thinning interval of 1. A fixed random seed (1520) was used to ensure reproducibility. Phasing accuracy was evaluated using posterior probabilities generated by PHASE. Site-specific phase probabilities were examined using the PHASEPROBS output, and the majority of SNPs showed posterior probabilities of 1.00. SNP positions with posterior probabilities ≥0.90 were considered confidently phased. Only a small proportion of sites exhibited posterior probabilities below this threshold, indicating low overall phasing uncertainty and high confidence in the inferred haplotypes. The results from both methods were compared to ensure the absence of any discrepancies.

The haplotypes identified in the Indonesian populations were aligned with the standard MHC-B haplotypes of heritage breeds and elite commercial egg layer lines [16,19] as well as Asian haplotypes from South Korea [17], Sri Lanka [18], Bangladesh [22], and Vietnam [23]. The alignment was performed using Clustal Omega and the phylogenetic trees were generated using the MrBayes approach (model HKY85) in Geneious Prime software (Geneious Prime 2024.0.7). Haplotypes were designated with the prefix “BSNP” to indicate that they were based on MHC-B SNPs and novel haplotypes were further labeled with “IND” to signify their origin in Indonesia. If a haplotype matched the standard haplotype across all 90 SNPs, it was labeled as standard. Otherwise, new names were assigned to novel haplotypes, with the numbering reflecting the sequence in which they were discovered (e.g., BSNP-IND01, BSNP-IND02, etc.) (Supplement 1). Observed (Ho) and expected heterozygosity (He) were calculated for each population using standard population genetic formulas implemented in R.

## RESULTS AND DISCUSSION

### Diversity of major histocompatibility complex-B single-nucleotide polymorphism haplotypes within and between Indonesian native chicken populations

**Figure 2:**
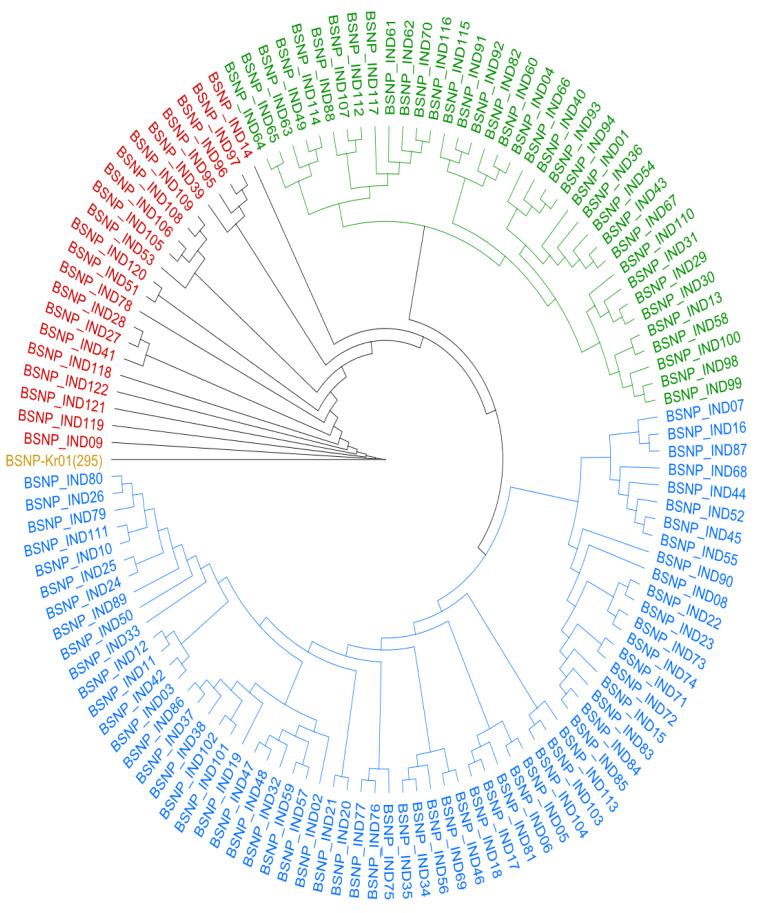
Bayesian approach-based circular cladogram illustrating the relationships among Indonesian BSNP haplotypes, with BSNP_Kr01 used as the outgroup. Colors indicate the identified subclades: red (Subclade 1), green (Subclade 2), and blue (Subclade 3).

High genetic diversity was observed using the MHC-B SNP panel (Table 1), with a total of 126 MHC-B haplotypes were identified across six Indonesian native chicken populations. The number of haplotypes ranged from 11 in the NNK population to 38 in the MRG population, resulting in an overall average of 21 haplotypes per population. All haplotypes discovered in this study were unique to the Indonesian native chicken populations, with the highest percentage similarity being 50% against the standard haplotypes reported by Fulton et al [19]. Three haplotypes were shared among populations: BSNP-IND14 was shared by the KJM and STL populations; BSNP-IND26 was shared by the KJM, NNK, and MRG populations; the KJM and MRG Populations shared BSNP-IND31 (Figure 2). Notably, all haplotypes in the GAG and PLG populations were unique. The observed heterozygosity (Ho) values ranged from 0.857 in the NNK population to 1.000 in the PLG population, while the expected heterozygosity (He) values varied from 0.666 in GAG to 0.966 in MRG (Table 1).

No distinctive major clades were observed in the phylogenetic analysis. Nevertheless, the identified Indonesian MHC-B haplotypes can be divided into three subclades (Figure 2). The networks were colour-coded to differentiate between different subclades. All six populations were represented in each subclade. Although the origin of the breeds is from different islands in Indonesia, the mixed clustering shows the possible human-mediated movement during the colonization period or the transmigration people program from one island to another by the Indonesian government. Similarly, hierarchical clustering of principal component analysis by Tribudi et al [25] reported that MRG and NNK were grouped as one subcluster, showing that although the populations originated from different islands that human-mediated movement played a role in the mixing of these populations. Furthermore, genetic relationships based on microsatellite DNA polymorphisms of Kampung, PLG, STL, and KJM revealed that these local chickens have the same ancestor. Kampung and STL were clustered together, followed by KJM and PLG [21], which further confirmed the dispersed nature of the populations despite the different locations of origin. In addition, Ramezani et al [26] demonstrated that MHC-linked microsatellite LEI0258 variability showed that the LEI0258 alleles from Indonesian chickens clustered into two groups: one private cluster comprising STL, GAG, and NNK breeds and another cluster where MRG and PLG grouped together, while KJM was positioned alongside Iranian and Tanzanian ecotypes. Moreover, Sulandari et al [27] studied 16 Indonesian chicken populations, including KJM, MRG, PLG, and STL, consisting of 335 mitochondrial D-loop sequences. Their analysis revealed that the phylogenetic relationships among these populations are not linked to their geographical locations. Furthermore, strong evolutionary forces, particularly pathogen-mediated balancing selection and local adaptation, are likely responsible for maintaining highly distinct MHC-B haplotypes within each chicken population despite potential gene flow among islands. Polymorphism in the MHC-B locus is frequently maintained by pathogen-driven balancing selection, which promotes the retention of multiple alleles to recognize a range of antigens [28]. In addition, distinct and locally adapted haplotypes are probably maintained by variable selection pressures imposed by various ecological conditions and pathogen [8,29]. Such conditions promote the retention of particular MHC-B haplotypes that are best suited to cope with the unique disease challenges of each environment. Consequently, even if human-mediated movement facilitates genetic exchange, directional selection in local contexts may counteract homogenization, allowing populations to remain genetically similar at neutral loci while continuing to exhibit marked divergence at adaptive immune genes such as MHC-B.

Collectively, these studies highlight that Indonesian native chicken populations are characterized by a dispersed genetic structure shaped more by human-mediated movement, admixture, and the pathogen pressure, rather than geographical origin.

### Comparison of major histocompatibility complex-B single-nucleotide polymorphism haplotypes in Indonesian native chickens with the major histocompatibility complex-B single-nucleotide polymorphism standard haplotypes

**Figure 3:**
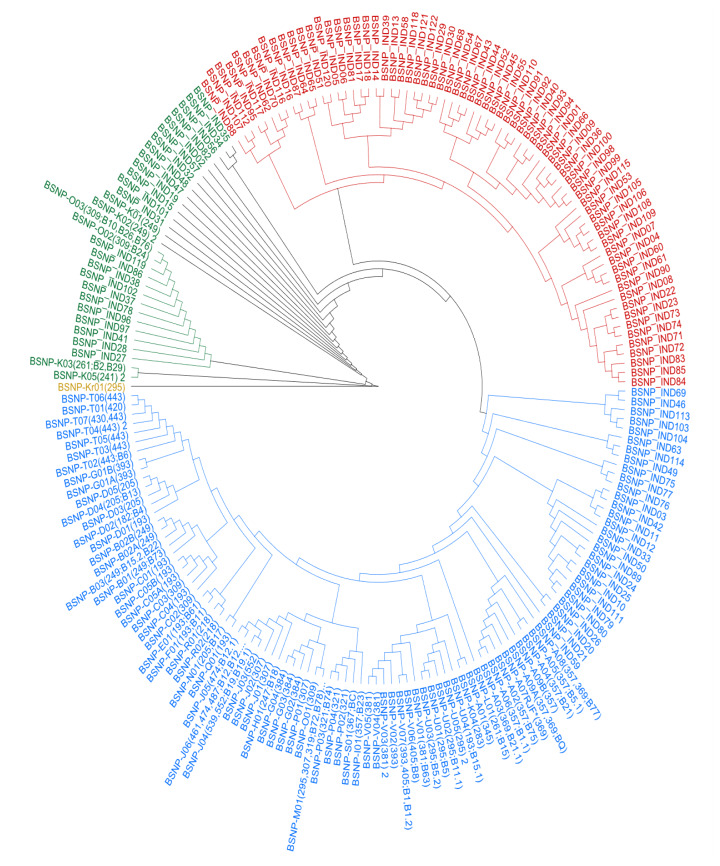
Bayesian approach-based circular cladogram illustrating genetic relationships among Indonesian haplotypes and MHC-B standard haplotypes, with BSNP_Kr01 used as the outgroup. Colors indicate the identified subclades: green (Subclade 1), red (Subclade 2), and blue (Subclade 3). MHC, major histocompatibility complex.

Comparison of MHC-B haplotypes in Indonesian native chickens with MHC-B standard haplotypes shows three possible subclades. Interestingly, except for one clade, the others show clustering of Indonesian MHC-B haplotypes with standard haplotypes (Figure 3). Some of these MHC-B standard haplotypes were also associated with certain B blood groups (serotypes) in chickens with known immune responses against diseases [8,10–14,30–33]. Despite this, none of the Indonesian native chickens’ MHC-B haplotypes exactly matched the standard haplotypes, confirming their novelty and suggesting the potential presence of distinct serotypes. Furthermore, this is the first study to reveal the MHC-B diversity in Indonesian native chickens using the MHC-B SNP panel. However, given that only six populations were included in the analysis, it remains possible that a complete match with standard MHC-B haplotypes may be identified in other untested populations. Leroy et al [34] reported that Indonesian native chickens retain some genetic characteristics from commercial chicken breeds and have not entirely replaced their genetic makeup.

### Comparison of major histocompatibility complex-B single-nucleotide polymorphism haplotypes in Indonesian native chickens with the major histocompatibility complex-B single-nucleotide polymorphism standard and Asian haplotypes

**Figure 4:**
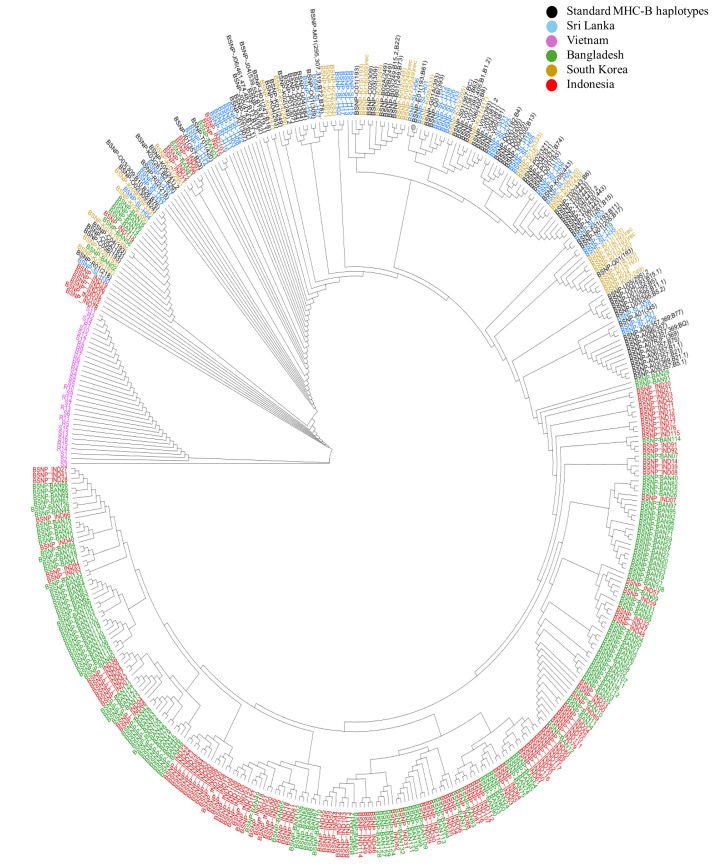
Bayesian approach-based circular cladogram illustrating genetic relationships among MHC-B haplotypes from standard references and Asian chicken populations, including Sri Lanka, Vietnam, Bangladesh, South Korea, and Indonesia. MHC, major histocompatibility complex.

Comparison of Indonesian MHC-B haplotypes with other Asian and standard haplotypes reveals that the majority of Indonesian haplotypes cluster with those from Bangladesh (Figure 4). Although the current study focuses on MHC-B SNP haplotype diversity, phylogenetic evidence from mtDNA D-loop sequences [35] reveals that Bangladeshi Red Junglefowls are closely related to Indonesian fowls (*G. g. bankiva,* distributed over Sumatra, Java, and Bali in Indonesia). Shared ancestry among South and Southeast Asian chicken populations could partly explain the observed patterns of MHC-B haplotype distribution in this study. However, after analyzing the complete mtDNA D-loop region, Oka et al [36] identified seven haplogroups and observed that Chinese and Korean chickens are derived from Southeast Asia, contradicting the findings of this study. Differences in genetic inheritance, evolutionary pressure, and historical demography can explain the above finding.

Moreover, Indonesia and Bangladesh both trace the ancestry of their chickens to *Gallus gallus spadiceus,* first domesticated in Thailand, Myanmar, and South China. According to Wang et al [37], Indonesian chickens show a mixture of *G. g. bankiva* and *G. g. gallus,* while South Asian populations, including Bangladesh, show admixture with *G. g. murghi*. The above results place Bangladesh and Indonesia as the endpoints of the ancient domesticated diffusion pathway from Southeast to South Asia, indicating a shared ancestral origin with region-specific gene flow. Moreover, it is evident that Southeast Asian maternal haplotypes have dispersed widely [38], maybe due to human-mediated movement; nevertheless, the extent and timing of such dispersal may differ from the gene flow patterns reflected in MHC-B. Compared with standard and global reference haplotypes, the highly divergent MHC-B haplotypes found in native Indonesian chickens indicate both evolutionary divergence and possible functional uniqueness. Private or novel alleles have been found in the BLB2 and LEI0258 loci in Indonesian native chickens, indicating adaptation to local pathogen pressures [24,39]. These genetic profiles may underline immunological traits that confer enhanced resistance to local pathogens. BLB2 correlates with enhanced antibody responses, including elevated IgY concentrations and resistance to diseases such as Newcastle Disease, suggesting functional immune benefits [39].

Several limitations should be considered when interpreting the results. Firstly, the limited number of samples may reduce the ability to detect already identified MHC-B haplotypes. Furthermore, due to a lack of disease challenge data, the functional association between the identified Indonesian MHC-B haplotypes and disease resistance could not be evaluated. Despite these limitations, this study provides valuable baseline information on MHC-B haplotype diversity in Indonesian native chickens.

## CONCLUSION

Evaluation through the MHC-B SNP panel reveals that Indonesian populations exhibit diverse and unique MHC-B haplotypes, which may reflect their potential adaptation to various pathogens. This MHC-B genetic diversity facilitates the identification of possible associations between specific MHC-B haplotypes and responses to diseases by future field-based disease challenge studies, thereby paving the way for selective breeding strategies that aim to enhance disease resistance in Indonesian native chickens.

## Figures and Tables

**Table 1:** The MHC-B haplotype results for Indonesian native chicken breeds

Population	No. samples^1)^	Total haplotype^2)^	Common haplotype^3)^	Unique haplotype^4)^	No. hom	No. het	Ho	He
Merawang (MRG)	24	38	2	36	1	23	0.958	0.966
Pelung (PLG)	17	25	0	25	0	17	1.000	0.947
Black Kedu (KJM)	30	19	3	16	3	27	0.900	0.872
Sentul (STL)	16	21	1	20	2	14	0.875	0.926
Nunukan (NNK)	14	11	1	10	2	12	0.857	0.875
Gaga (GAG)	20	12	0	12	1	19	0.950	0.666

1) Number of samples per population used for haplotype analysis.

2) Number of haplotypes identified in each population.

3) Number of common haplotypes per population.

4) Unique haplotypes observed in each population.

No. hom, number of homozygous birds for MHC-B haplotypes; No. het, number of heterozygous birds for MHC-B haplotypes; Ho, observed heterozygosity; He, expected heterozygosity; MHC-B, major histocompatibility complex B.

## Data Availability

The complete set of MHC-B haplotypes identified in Indonesian native chickens is available in Supplement 1.

## SUPPLEMENTARY MATERIAL

**Supplement 1.** MHC-B haplotypes identified in Indonesian native chickens.
