# Supplementary material for: Identification and comparative analysis of novel major histocompatibility complex-B haplotypes in Indonesian native chickens
Source: Anim Biosci. 2026 Mar 11;39(6):250842. doi: 10.5713/ab.250842 (PMC13243977; doi:10.5713/ab.250842)
Supplement: Supplementary file 1 [file ab-250842-Supplementary-1.pdf]

Supplement 1. MHC-B haplotypes identified in Indonesian native chickens
MHC-B SNP panel

Haplotype	MHC-J6	MHC-5	MHCNew20	MHCNew21	MHCNew22	MHC-8	MHCNew24	MHC-9	MHC-11	MHCNew25	MHCNew26	MHC-12	MHC-14	MHC-15	MHCNew28	MHC-18	MHC-25	MHC-26	MHC-30	MHC-31	MHC-33	MHC-39	MHC-48	MHC-49	MHC-53	MHC-54	MHC-56	MHC-57	MHC-58	MHC-60	MHC-61	MHC-63	MHC-64	MHC-65	MHC-67	MHC-68	MHC-69	MHC-70	MHC-71	MHC-72
BSNP_IND01	T	T	G	A	T	A	A	T	T	T	G	C	T	C	A	A	G	T	T	T	C	T	C	T	A	C	A	A	T	G	G	T	T	T	C	C	C	C	A	G
BSNP_IND02	T	T	T	G	T	A	A	T	T	T	G	T	T	C	A	G	G	T	T	T	G	T	C	T	A	C	A	A	T	G	G	T	T	C	C	C	C	C	A	G
BSNP_IND03	T	T	G	A	T	A	A	T	C	T	G	C	T	C	G	A	G	T	T	T	G	T	C	T	A	C	A	A	T	G	G	T	T	C	C	C	C	C	A	G
BSNP_IND04	T	T	G	A	T	A	A	T	T	T	G	T	T	C	G	G	G	T	T	T	C	T	C	T	A	C	A	G	C	T	G	T	T	T	C	C	T	C	A	G
BSNP_IND05	T	T	T	A	C	G	A	T	C	C	G	C	T	C	A	A	A	T	T	C	G	T	C	T	A	C	A	A	T	G	G	C	T	C	T	C	C	C	A	G
BSNP_IND06	T	T	T	A	T	A	A	T	C	T	G	C	T	C	G	A	A	T	T	T	G	T	C	T	G	C	A	A	T	G	G	T	T	C	C	C	T	C	A	G
BSNP_IND07	T	T	T	A	T	A	A	T	T	T	G	C	T	C	A	A	A	T	T	T	G	T	C	T	A	C	A	A	T	G	G	T	C	C	C	C	C	C	A	G
BSNP_IND08	T	T	T	A	T	A	A	T	C	C	G	T	T	C	A	G	G	T	T	C	C	T	T	T	A	C	A	A	T	G	G	T	C	C	C	C	T	C	A	G
BSNP_IND09	T	T	T	A	T	A	A	T	C	C	G	T	T	C	A	G	G	T	T	C	C	T	T	T	A	C	A	A	T	G	T	T	C	C	C	C	T	C	A	G
BSNP_IND10	T	T	G	A	T	A	A	T	C	T	G	C	T	C	A	A	G	T	T	C	C	T	T	T	A	C	A	A	T	G	G	T	C	C	C	C	T	C	A	G
BSNP_IND11	T	T	G	A	C	A	A	T	T	T	G	C	T	C	G	A	G	T	T	T	C	T	T	T	G	C	A	A	T	G	G	T	T	C	C	C	T	C	A	G
BSNP_IND12	T	T	G	A	C	G	A	T	T	C	G	C	T	C	A	A	A	T	T	T	C	T	T	T	G	C	A	A	T	G	T	T	T	C	T	C	C	T	A	A
BSNP_IND13	T	T	T	A	T	A	A	T	C	C	G	T	T	C	A	G	G	T	T	T	C	T	T	T	G	C	A	A	C	T	T	T	T	C	C	T	T	C	A	A
BSNP_IND14	T	T	T	A	T	A	A	T	C	T	G	T	T	C	G	G	G	T	T	T	C	C	T	T	G	G	A	A	C	T	G	T	T	C	C	T	T	C	G	G
BSNP_IND15	T	T	T	A	T	G	A	T	C	C	A	T	T	C	A	A	G	T	T	T	C	T	T	T	A	C	A	A	T	G	G	T	C	C	C	C	T	C	A	G
BSNP_IND16	T	T	T	A	T	G	A	T	C	C	A	T	T	C	A	A	G	T	T	T	C	T	T	T	A	C	A	A	T	G	G	T	C	C	C	C	T	C	A	G
BSNP_IND17	T	T	G	A	T	A	A	T	C	C	G	C	T	C	A	A	G	T	T	T	C	T	T	T	A	C	A	A	T	G	G	T	C	C	C	C	C	C	A	A
BSNP_IND18	T	T	G	A	C	A	A	T	C	C	G	C	T	C	G	G	G	T	T	T	C	T	T	T	A	C	A	A	C	T	G	C	T	C	C	T	C	C	A	A
BSNP_IND19	T	T	G	A	C	A	A	T	C	C	G	C	T	C	G	G	G	T	T	T	C	T	T	T	A	C	A	A	C	T	G	C	T	C	C	T	C	C	A	A
BSNP_IND20	T	T	G	A	T	A	A	T	C	T	G	T	T	C	A	A	G	T	T	T	C	T	T	T	A	C	A	A	T	G	G	C	T	C	C	T	C	T	A	A
BSNP_IND21	T	T	G	A	T	A	A	T	T	C	G	T	T	C	G	A	G	T	T	C	C	T	C	T	A	C	A	A	T	G	G	C	T	C	C	T	C	C	A	G
BSNP_IND22	T	T	G	A	T	A	A	T	C	T	G	C	T	C	G	A	G	T	T	T	G	T	T	T	G	C	A	A	T	G	G	C	T	C	C	T	T	C	G	A
BSNP_IND23	T	T	T	A	T	G	A	T	C	C	A	C	T	G	A	A	G	T	T	T	G	T	T	T	G	C	A	A	T	G	G	C	T	C	C	C	T	C	G	G
BSNP_IND24	T	T	T	A	T	G	A	T	C	C	A	C	T	G	A	A	G	T	T	T	G	T	T	T	G	C	A	A	T	G	G	C	T	C	C	C	T	C	G	G
BSNP_IND25	T	T	T	A	C	A	A	T	C	T	G	C	T	C	A	A	G	T	T	T	G	T	C	T	A	C	A	A	T	G	G	T	T	C	C	C	C	C	A	G
BSNP_IND26	T	T	T	A	C	A	A	T	C	T	G	C	T	C	A	A	G	T	T	T	G	T	C	T	A	C	A	A	T	G	G	T	T	C	C	C	C	C	A	G
BSNP_IND27	T	T	G	A	C	G	A	T	C	C	G	T	T	C	A	G	G	T	T	T	C	T	T	T	A	C	A	A	T	G	G	C	T	C	T	C	T	C	A	G
BSNP_IND28	T	T	G	A	C	G	A	T	T	C	G	T	T	C	G	G	G	T	T	T	C	T	T	T	A	C	A	A	T	G	G	C	T	C	C	C	T	C	A	G
BSNP_IND29	T	T	T	A	C	A	A	G	C	C	G	T	T	C	A	G	G	T	T	T	C	T	T	T	A	G	A	A	T	G	G	T	T	C	T	C	T	C	A	G
BSNP_IND30	T	T	G	A	T	G	A	T	C	T	G	T	T	C	G	G	G	T	T	T	C	T	T	T	A	G	A	A	T	G	G	T	T	C	T	C	T	C	A	G
BSNP_IND31	T	T	G	A	C	A	A	G	T	C	G	T	T	C	A	A	G	T	A	T	C	T	T	T	A	G	A	A	T	G	G	T	T	C	T	C	C	C	A	G
BSNP_IND32	T	T	T	A	T	A	A	T	C	T	G	T	T	C	G	G	G	T	T	T	C	T	T	T	G	G	A	A	T	G	G	T	T	C	T	C	C	C	A	A
BSNP_IND33	T	T	T	A	T	A	A	T	C	T	G	T	T	C	G	G	G	T	T	T	C	T	T	T	G	G	A	A	T	G	T	T	T	C	T	C	C	C	A	A
BSNP_IND34	T	T	T	A	T	G	A	T	C	T	A	C	T	C	A	G	G	T	T	T	C	T	T	T	A	C	A	A	T	G	G	T	T	C	T	C	C	C	A	G
BSNP_IND35	T	T	T	A	T	G	G	T	T	T	A	C	T	C	A	G	G	T	T	T	C	T	T	T	A	C	A	A	T	G	G	T	C	C	T	C	C	C	A	G
BSNP_IND36	T	T	G	A	C	A	A	T	T	C	G	C	T	C	A	A	G	T	T	T	C	T	T	T	A	C	A	A	C	G	G	T	T	C	T	C	C	C	A	A
BSNP_IND37	T	T	G	A	T	A	A	T	C	T	G	T	T	G	G	G	G	T	T	C	C	T	T	T	A	C	A	A	T	G	G	C	T	C	C	T	T	C	G	G
BSNP_IND38	T	T	G	A	T	A	A	G	C	C	G	T	T	C	A	G	G	T	T	T	C	T	T	T	A	G	A	A	T	G	G	T	T	C	T	C	T	C	A	G
BSNP_IND39	T	T	G	A	T	G	A	T	T	T	A	T	T	C	A	A	A	T	T	C	C	T	T	T	A	C	A	A	T	G	G	T	T	C	C	T	T	C	A	A
BSNP_IND40	T	T	T	G	T	A	A	T	T	T	A	T	T	C	G	A	A	T	T	C	C	T	T	T	G	C	A	A	T	G	G	T	C	C	C	C	T	C	A	A
BSNP_IND41	T	T	T	A	T	G	A	T	C	T	G	T	T	C	A	G	A	T	T	C	C	T	T	T	G	C	A	A	T	G	G	T	T	C	T	T	T	C	G	A
BSNP_IND42	T	T	T	A	T	G	A	T	C	T	G	T	T	C	A	G	A	T	T	C	C	T	T	T	G	C	A	A	T	G	T	T	T	C	T	T	T	C	G	A
BSNP_IND43	T	T	G	A	C	A	A	T	T	T	G	C	T	C	A	A	G	T	T	T	C	T	T	T	A	C	A	A	T	T	G	T	T	C	T	T	C	C	A	A
BSNP_IND44	T	T	G	A	C	A	A	T	T	T	G	C	T	C	A	A	G	T	T	T	C	T	T	T	A	C	A	A	T	T	G	T	T	C	T	T	C	C	A	A
BSNP_IND45	T	T	G	A	C	A	G	T	C	T	G	T	T	C	G	A	G	T	T	C	C	T	T	T	A	C	G	A	T	G	G	C	T	C	T	T	T	C	A	G
BSNP_IND46	T	T	G	A	C	A	G	T	C	T	G	T	T	C	G	A	G	T	T	C	C	T	T	T	A	C	G	A	T	G	G	C	T	C	T	T	T	C	A	G
BSNP_IND47	T	T	G	A	C	A	A	T	C	T	G	T	T	C	A	A	G	T	A	T	C	T	T	T	G	G	A	A	T	G	G	T	T	C	T	C	T	C	A	G
BSNP_IND48	T	T	G	A	T	A	A	T	C	T	G	C	T	C	G	A	G	T	T	C	C	T	T	T	G	C	A	G	C	T	G	T	T	T	T	T	C	C	A	A
BSNP_IND49	T	T	T	A	T	A	A	T	C	T	G	C	T	C	G	A	A	T	T	T	G	T	T	T	G	C	A	G	C	T	G	T	T	T	T	T	C	C	A	A
BSNP_IND50	G	C	G	G	T	A	A	T	T	C	G	C	T	C	G	A	G	T	T	C	C	T	T	T	G	C	A	G	C	T	G	T	T	T	T	T	C	C	A	A
BSNP_IND51	G	C	G	G	T	A	A	T	T	C	G	C	T	G	A	A	G	T	T	T	C	T	T	T	G	C	A	A	C	T	G	T	T	C	C	T	C	C	A	A
BSNP_IND52	T	T	T	G	C	G	A	T	T	T	G	C	T	C	G	G	G	T	T	C	C	T	C	T	A	C	A	G	C	T	G	T	T	T	T	C	C	C	A	G
BSNP_IND53	T	T	T	G	C	G	A	T	T	T	G	C	T	C	G	G	G	T	T	C	C	T	C	T	A	C	A	G	C	T	G	T	T	T	T	C	C	C	A	G
BSNP_IND54	T	T	T	G	C	G	A	T	T	T	G	C	T	C	G	G	G	T	T	C	C	T	C	T	A	C	A	G	C	T	G	T	T	T	T	C	C	C	A	G
BSNP_IND55	T	T	T	G	C	G	A	T	T	T	G	C	T	C	G	G	G	T	T	C	C	T	C	T	A	C	A	G	C	T	G	T	T	T	T	C	C	C	A	G
BSNP_IND56	T	T	T	G	C	G	A	T	T	T	G	C	T	C	G	G	G	T	T	C	C	T	C	T	A	C	A	G	C	T	G	T	T	T	T	C	C	C	A	G
BSNP_IND57	T	T	T	G	C	G	A	T	T	T	G	C	T	C	G	G	G	T	T	C	C	T	C	T	A	C	A	G	C	T	G	T	T	T	T	C	C	C	A	G
BSNP_IND58	T	T	T	G	C	G	A	T	T	T	G	C	T	C	G	G	G	T	T	C	C	T	C	T	A	C	A	G	C	T	G	T	T	T	T	C	C	C	A	G
BSNP_IND59	T	T	G	A	C	A	A	T	T	T	G	C	T	C	G	G	G	T	T	C	C	T	C	T	A	C	A	G	C	T	G	T	T	T	T	C	C	C	A	G
BSNP_IND60	T	T	T	G	T	A	A	T	T	T	G	C	T	C	A	A	G	T	T	C	C	T	T	T	G	C	G	G	C	T	G	T	T	T	T	C	C	C	A	G
BSNP_IND61	T	T	T	A	T	G	A	T	T	T	G	C	T	C	A	G	G	T	A	C	C	T	T	T	A	C	A	G	C	T	G	T	T	T	T	T	C	C	A	A
BSNP_IND62	T	T	T	A	T	G	A	T	T	T	G	C	T	C	A	G	G	T	A	C	C	T	T	T	A	C	A	G	C	T	T	T	T	T	T	T	C	C	A	A
BSNP_IND63	T	T	T	A	T	G	A	T	T	T	G	T	T	C	G	G	G	T	T	T	C	T	C	T	A	C	G	G	C	T	G	T	T	T	C	T	T	C	A	G
BSNP_IND64	T	T	G	A	T	A	A	G	C	T	G	C	T	C	A	A	G	T	T	T	C	T	C	T	A	C	G	G	C	T	G	T	T	T	C	T	T	C	A	G
BSNP_IND65	T	T	G	A	T	A	A	G	C	T	G	C	T	C	A	A	G	T	T	T	C	T	C	T	A	C	G	G	C	T	G	T	T	C	C	T	T	C	A	G
BSNP_IND66	T	T	G	A	T	A	A	G	C	T	G	C	T	C	A	A	G	T	T	T	C	T	C	T	A	C	G	G	C	T	G	T	T	T	C	T	T	C	A	G
BSNP_IND67	G	C	G	G	T	A	A	T	T	C	G	C	T	C	G	A	G	T	T	T	C	T	C	T	A	C	G	G	C	T	T	T	T	C	C	T	T	C	A	G
BSNP_IND68	G	C	G	G	T	A	A	T	C	C	G	C	T	C	G	A	G	T	T	T	C	T	C	T	A	C	G	G	C	T	G	T	T	C	C	T	T	C	A	G
BSNP_IND69	T	T	T	A	T	A	A	T	T	C	G	T	T	C	A	G	A	T	T	T	C	T	C	T	A	C	G	G	C	T	G	T	T	T	C	C	T	C	A	G
BSNP_IND70	T	T	T	A	T	A	A	T	T	C	G	T	T	C	A	G	A	T	T	T	C	T	C	T	A	C	G	G	C	T	G	T	T	T	C	C	T	C	A	G
BSNP_IND71	T	T	T	A	T	A	A	T	T	C	G	T	T	C	A	G	A	T	T	T	C	T	C	T	A	C	G	G	C	T	G	T	T	T	C	C	T	C	A	G
BSNP_IND72	T	T	G	A	T	G	A	T	C	T	G	C	T	C	A	A	G	T	T	T	G	T	C	T	A	C	G	A	T	G	G	T	T	C	T	C	C	C	A	G
BSNP_IND73	T	T	G	A	T	A	A	G	C	T	G	C	T	C	A	A	G	T	T	T	C	T	C	T	A	C	A	G	C	T	G	T	T	C	C	C	T	C	A	G
BSNP_IND74	T	T	G	A	T	G	A	T	T	T	G	T	T	C	G	G	G	T	T	T	C	T	C	T	A	C	G	G	C	T	G	T	T	C	C	T	T	C	A	G
BSNP_IND75	T	T	G	A	T	A	A	T	C	T	G	T	T	C	G	G	G	T	T	T	C	T	C	T	A	C	A	A	T	T	G	T	T	T	T	C	C	C	A	A
BSNP_IND76	T	T	G	A	C	A	A	G	C	C	G	T	T	C	A	G	G	T	T	T	C	T	T	T	A	G	A	A	T	G	G	T	C	C	T	C	T	C	A	A
BSNP_IND77	T	T	G	A	C	A	A	G	C	C	G	T	T	C	A	G	G	T	T	T	C	T	T	T	A	G	A	A	T	G	T	T	C	C	T	C	C	C	A	A
BSNP_IND78	G	C	G	G	T	A	A	T	T	C	G	C	T	C	G	G	A	T	T	C	C	T	T	T	A	C	A	A	T	G	G	T	T	C	C	C	T	C	A	G
BSNP_IND79	G	C	G	G	T	A	A	T	T	C	G	C	T	C	G	G	A	T	T	C	C	T	T	T	A	C	A	A	T	G	T	T	T	C	C	C	T	C	A	G
BSNP_IND80	G	C	G	G	T	A	A	T	T	C	G	C	C	G	A	A	G	T	T	T	C	T	T	T	G	C	G	A	T	T	G	C	T	C	C	T	T	C	G	A
BSNP_IND81	T	T	T	G	T	A	A	T	C	T	G	C	T	C	G	G	A	T	T	T	C	T	T	T	A	C	G	A	T	G	G	T	C	C	T	C	C	C	A	A
BSNP_IND82	T	T	G	G	C	A	A	T	C	C	G	C	T	C	A	A	G	T	T	T	C	T	T	T	A	C	A	A	T	T	G	T	C	C	C	T	T	C	G	A
BSNP_IND83	T	T	T	A	C	G	A	G	T	C	G	C	T	C	G	A	G	T	T	T	C	T	T	T	A	C	A	A	T	T	G	T	T	T	T	C	T	C	A	A
BSNP_IND84	T	T	T	A	C	G	A	G	T	C	G	C	T	C	G	A	G	T	T	T	C	T	T	T	A	C	A	A	T	T	G	T	T	T	T	C	T	T	A	A
BSNP_IND85	T	T	T	A	C	G	G	G	T	C	G	C	T	C	G	A	G	T	T	T	C	T	T	T	A	C	A	A	T	T	G	T	T	T	T	C	T	C	A	A
BSNP_IND86	T	T	T	G	T	A	A	G	C	T	G	C	T	C	A	A	G	T	T	T	C	T	T	T	A	C	A	A	T	G	G	T	C	T	C	C	C	C	A	A
BSNP_IND87	T	T	G	G	C	A	A	G	C	T	G	C	T	C	G	A	G	T	T	T	C	T	T	T	G	C	A	A	C	T	T	T	T	C	C	T	T	C	A	A
BSNP_IND88	T	T	T	A	T	A	A	T	T	T	G	C	T	C	G	A	G	T	T	C	C	T	C	T	A	C	A	A	T	G	G	T	T	C	T	T	T	C	G	A
BSNP_IND89	T	T	G	A	T	A	A	T	T	T	G	T	T	C	A	G	G	T	T	T	C	T	T	T	G	C	A	A	C	T	T	T	T	C	C	T	T	C	A	A
BSNP_IND90	T	T	G	A	T	A	A	T	C	T	G	C	T	C	A	G	G	T	T	T	C	T	T	T	G	C	A	A	C	T	T	T	T	C	C	T	T	C	A	A
BSNP_IND91	T	T	G	A	T	A	A	T	C	T	G	C	T	C	A	G	G	T	T	T	C	T	T	T	G	C	A	A	C	T	T	T	T	C	C	T	T	C	A	A
BSNP_IND92	T	T	G	A	T	A	A	T	C	T	G	C	T	C	A	G	G	T	T	T	C	T	T	T	G	C	A	A	C	T	T	T	T	C	C	T	T	T	A	A
BSNP_IND93	T	T	T	A	T	A	A	T	T	T	G	C	T	C	G	A	A	T	A	T	G	T	T	T	A	C	A	G	C	T	G	T	T	T	T	C	C	C	A	G
BSNP_IND94	T	T	G	A	C	A	G	T	T	C	G	C	T	C	G	A	G	G	T	C	C	T	T	T	G	C	A	A	T	G	T	T	T	C	C	T	T	T	A	G
BSNP_IND95	T	T	G	A	C	G	A	T	C	T	G	T	T	C	A	A	A	T	A	C	G	C	T	T	G	G	A	A	T	G	G	T	T	C	C	T	C	C	A	G
BSNP_IND96	G	C	G	G	T	A	A	T	T	C	G	C	T	C	A	A	A	T	T	C	G	C	T	T	G	G	A	A	T	G	G	T	T	C	C	T	C	C	A	G
BSNP_IND97	G	C	G	G	T	A	G	T	T	C	G	C	T	G	A	A	A	T	T	C	G	C	T	T	G	G	A	A	T	G	G	T	T	C	C	T	C	C	A	G
BSNP_IND98	T	T	G	A	T	G	A	T	T	T	G	T	T	C	A	A	A	T	A	T	C	T	T	T	A	C	G	A	T	G	G	T	T	C	C	T	T	C	G	A
BSNP_IND99	T	T	G	A	C	A	A	T	T	C	G	C	C	G	A	A	A	T	T	T	C	T	T	T	A	C	G	A	T	G	G	T	C	C	C	T	T	C	A	G
BSNP_IND100	T	T	G	A	T	A	A	T	C	C	G	T	T	C	A	G	G	T	T	C	C	T	T	T	A	C	A	A	T	G	G	T	C	C	C	C	T	C	A	G
BSNP_IND101	T	T	T	A	T	A	A	T	T	T	G	C	T	C	G	A	G	T	T	T	C	T	T	T	A	C	A	A	T	G	G	T	C	C	C	T	T	C	A	A
BSNP_IND102	T	T	G	A	C	A	A	T	C	C	G	C	T	C	G	A	G	T	T	T	C	T	T	T	A	C	G	A	T	G	G	C	C	C	T	C	C	C	A	G
BSNP_IND103	T	T	G	A	C	A	A	T	C	C	G	C	T	C	G	A	G	T	T	T	C	T	T	T	A	C	G	A	T	G	G	C	C	C	T	C	C	C	A	G
BSNP_IND104	T	T	G	A	T	A	A	T	C	T	G	T	T	C	A	G	G	T	T	T	C	T	T	T	A	C	A	A	T	G	G	T	C	C	C	C	T	C	A	G
BSNP_IND105	T	T	G	A	T	A	A	T	T	T	G	T	T	C	G	G	G	T	T	T	C	T	T	T	G	C	A	A	T	G	G	T	C	C	C	C	T	C	A	G
BSNP_IND106	T	T	G	A	C	A	A	T	C	T	G	T	T	C	A	G	G	T	T	T	C	T	T	T	A	C	G	A	T	G	G	T	T	C	C	T	T	C	G	A
BSNP_IND107	T	T	G	A	C	A	A	T	T	T	G	C	T	C	A	A	G	T	T	T	C	T	T	T	A	C	A	A	T	G	G	T	T	C	C	T	T	C	G	A
BSNP_IND108	T	T	G	A	C	A	A	T	T	T	G	C	T	C	A	A	G	T	T	T	C	T	T	T	A	C	A	A	T	G	G	T	T	C	C	C	C	C	A	G
BSNP_IND109	T	T	T	A	T	A	A	T	C	T	G	C	T	C	G	A	G	T	T	T	C	T	T	T	G	C	A	A	T	G	T	T	T	C	C	C	T	C	A	G
BSNP_IND110	T	T	G	A	C	A	A	T	T	T	G	C	T	C	G	A	G	T	T	T	C	T	T	T	G	C	A	A	T	G	G	T	T	C	C	C	T	C	A	G
BSNP_IND111	T	T	G	A	C	A	A	T	T	T	G	C	T	C	G	A	G	T	T	T	C	T	T	T	A	C	A	A	T	G	G	T	C	C	C	T	T	T	G	A
BSNP_IND112	T	T	T	A	C	A	A	T	T	C	G	C	T	C	G	G	G	T	T	C	C	T	T	T	A	C	A	A	T	G	G	T	C	C	T	T	T	C	G	A
BSNP_IND113	T	T	G	A	T	A	A	T	T	T	G	C	T	C	G	G	G	T	T	C	C	T	T	T	A	C	A	A	T	G	G	T	C	C	T	T	T	C	G	A
BSNP_IND114	T	T	G	A	T	G	A	T	T	T	G	C	T	C	A	G	A	T	T	T	C	T	T	T	G	C	A	A	C	G	G	T	T	C	T	T	T	C	A	G
BSNP_IND115	T	T	G	A	T	G	A	T	T	T	G	C	T	C	A	G	A	T	T	T	C	T	T	T	G	C	A	A	T	G	G	T	T	C	T	T	T	C	A	G
BSNP_IND116	G	C	G	G	T	A	A	T	T	C	G	C	T	C	A	A	G	T	T	T	C	T	T	T	A	C	A	A	C	T	G	T	C	C	T	C	C	C	G	A
BSNP_IND117	G	C	G	G	T	A	A	T	T	C	G	C	T	C	A	A	G	T	T	T	C	T	T	T	A	C	A	A	C	T	G	T	C	C	T	C	C	C	G	A
BSNP_IND118	G	C	G	G	T	A	A	T	C	C	G	T	T	C	A	A	G	T	T	T	C	T	T	T	A	C	A	A	C	T	G	T	C	C	T	C	C	C	G	A
BSNP_IND119	T	T	G	A	C	G	A	T	T	C	G	C	T	C	A	A	A	T	T	T	C	T	T	T	G	C	A	A	T	G	G	T	T	C	T	C	C	T	A	A
BSNP_IND120	T	T	G	A	C	G	A	T	T	C	G	C	T	C	A	A	A	T	T	T	C	T	T	T	G	C	A	A	C	T	G	T	T	C	T	C	C	T	A	A
BSNP_IND121	T	T	T	G	T	A	A	T	T	T	G	T	T	C	A	G	G	T	T	T	C	T	T	T	A	C	A	A	C	T	G	T	C	C	C	T	T	C	A	A
BSNP_IND122	T	T	T	G	T	A	A	T	T	T	G	T	T	C	A	G	G	T	T	T	C	T	T	T	A	C	A	A	C	T	G	T	C	C	C	T	T	C	A	A

Haplotype	MHC-75	MHC-77	MHC-79	MHC-80	MHC-81	MHC-83	MHC-84	MHC-85	MHC-87	MHC-89	MHC-91	MHC-92	MHC-94	MHC-96	MHC-97	MHC-99	MHC-101	MHC-102	MHC-105	MHC-106	MHC-111	MHC-114	MHC-116	MHC-118	MHC-119	MHC-120	MHC-124	MHC-127	MHC-129	MHC-134	MHC-135	MHC-137	MHC-138	MHC-139	MHC-142	MHC-144	MHC-150	MHC-151	MHC-155	MHC-156	MHC-157	MHC-160	MHC-162	MHC-167	MHC-168	MHC-169	MHC-170	MHC-171	MHC-173	MHC-178
BSNP_IND01	G	C	G	C	C	C	A	C	T	T	T	T	A	T	T	C	C	C	C	C	A	A	C	A	A	T	A	G	G	G	A	A	G	A	T	A	T	A	A	A	A	A	T	G	A	G	T	C	T	C
BSNP_IND02	G	C	A	C	T	C	A	C	T	T	T	C	A	T	G	C	C	C	C	C	A	A	C	A	A	C	A	G	G	G	A	A	G	A	T	A	T	A	A	A	A	A	T	G	G	G	T	C	T	C
BSNP_IND03	G	C	A	T	T	C	A	C	T	T	T	T	G	T	G	C	C	C	C	T	G	A	C	A	A	C	A	G	G	G	A	A	G	A	T	A	T	A	A	G	A	A	T	G	G	G	T	C	T	C
BSNP_IND04	G	C	G	T	C	C	A	C	T	T	T	T	G	T	T	C	T	C	C	C	A	A	C	A	A	T	A	G	G	G	G	A	G	G	T	A	T	A	A	A	G	A	T	G	A	G	C	T	T	C
BSNP_IND05	G	C	A	C	T	C	A	C	T	T	C	C	A	T	G	C	C	C	C	T	A	A	C	A	G	C	A	A	A	G	A	A	G	A	T	A	T	A	A	A	A	A	T	G	G	G	T	C	T	C
BSNP_IND06	G	C	A	T	T	C	A	C	T	T	T	T	G	G	G	C	C	T	C	T	G	A	C	A	G	C	G	A	G	G	A	G	C	A	T	A	T	A	A	G	G	A	T	G	A	G	T	C	T	C
BSNP_IND07	G	C	A	C	T	C	G	C	T	T	T	T	G	G	T	C	T	T	C	T	G	A	C	A	A	T	G	G	G	G	A	G	C	A	T	A	T	A	A	G	G	A	T	G	A	G	T	C	T	C
BSNP_IND08	G	C	A	C	C	C	A	C	T	T	T	T	A	G	T	C	C	T	C	T	G	A	C	A	A	T	G	G	A	G	G	G	C	A	T	G	T	A	A	A	G	A	T	G	A	G	T	C	T	C
BSNP_IND09	G	C	A	C	C	C	A	C	T	T	T	C	A	G	T	C	C	T	C	T	G	A	C	A	A	T	A	G	G	G	A	A	G	A	T	G	T	A	A	G	G	A	T	G	A	A	C	T	C	C
BSNP_IND10	G	C	A	C	C	C	A	C	T	T	T	T	A	G	T	C	C	T	C	T	G	A	C	A	A	T	A	G	G	G	A	A	G	A	T	G	T	A	A	G	G	A	T	G	A	A	C	T	C	C
BSNP_IND11	G	T	G	T	C	C	A	C	T	T	T	T	G	T	G	C	C	C	C	T	A	A	C	A	G	C	A	A	A	C	A	A	G	G	T	A	T	A	A	A	A	A	T	G	A	A	C	T	C	C
BSNP_IND12	G	C	A	C	C	C	A	T	T	G	C	T	A	T	G	C	T	C	C	C	A	A	C	G	A	T	A	A	A	G	A	A	G	A	T	A	T	A	A	A	A	A	T	G	A	G	T	C	T	C
BSNP_IND13	G	C	A	T	C	C	A	C	T	T	T	T	A	T	G	C	T	T	C	T	A	A	C	A	A	T	A	A	A	C	G	A	G	A	T	G	T	A	A	A	G	A	T	G	A	G	T	C	T	C
BSNP_IND14	G	C	A	C	C	C	A	C	T	T	T	C	A	T	G	C	C	T	C	T	A	A	C	A	G	C	A	G	A	C	G	A	G	A	T	G	T	A	A	A	G	A	T	G	A	A	C	C	T	C
BSNP_IND15	G	C	A	T	T	C	A	C	T	T	T	T	A	T	G	C	T	T	C	T	A	A	C	A	A	T	A	A	A	G	G	A	G	A	T	G	T	A	A	G	G	A	T	G	A	A	C	T	C	G
BSNP_IND16	A	C	A	T	T	C	A	C	T	T	T	T	A	T	G	C	T	T	C	T	A	A	C	A	A	T	A	G	A	G	G	A	G	A	T	G	T	A	A	G	G	A	T	G	A	A	C	T	C	G
BSNP_IND17	G	C	A	T	C	C	A	C	T	T	T	T	A	T	G	C	T	T	C	T	A	A	C	A	A	T	A	A	A	C	G	A	G	A	T	G	T	A	A	G	G	A	T	G	A	A	C	T	T	C
BSNP_IND18	G	T	G	T	C	C	A	C	T	G	C	T	G	T	T	C	T	C	C	T	A	A	C	A	A	T	A	G	A	G	A	A	C	A	T	G	T	A	A	A	G	A	T	G	A	A	C	C	T	C
BSNP_IND19	G	C	G	T	C	C	A	C	T	G	C	T	G	T	T	C	T	C	C	T	A	A	C	A	A	T	A	G	A	G	A	A	C	A	T	G	T	A	A	A	G	A	T	G	A	A	C	C	T	C
BSNP_IND20	G	C	G	T	C	C	A	C	T	G	C	T	G	T	T	C	T	C	C	T	A	A	C	A	A	T	A	G	A	G	A	A	C	A	T	G	T	A	A	A	G	A	T	G	A	A	C	C	T	C
BSNP_IND21	G	C	A	T	C	C	A	C	T	G	C	T	A	G	T	C	T	C	C	T	A	G	C	A	A	T	A	A	A	G	A	A	C	A	T	A	T	A	A	A	A	A	T	G	A	G	C	C	T	C
BSNP_IND22	G	C	A	T	C	C	A	C	T	T	C	T	A	G	T	T	C	C	C	T	G	G	C	A	A	T	A	A	A	G	G	A	G	A	T	A	T	A	A	A	G	A	T	G	A	G	T	C	T	C
BSNP_IND23	G	T	A	T	C	C	A	C	T	T	C	T	A	G	T	T	C	C	C	T	G	G	C	A	A	T	A	A	A	G	G	A	G	A	T	A	T	A	A	A	A	A	A	G	A	A	C	C	C	C
BSNP_IND24	G	C	A	T	C	C	A	C	T	T	C	T	A	G	T	T	C	C	C	T	G	G	C	A	A	T	A	A	A	G	G	A	G	A	T	A	T	A	A	A	A	A	A	G	A	A	C	C	C	C
BSNP_IND25	G	T	A	T	C	C	A	C	T	T	C	T	A	G	T	T	C	C	C	T	G	G	C	A	A	T	A	A	A	G	G	A	G	A	T	A	T	A	A	A	A	A	A	G	A	A	C	C	C	C
BSNP_IND26	G	C	A	T	C	C	A	C	T	T	C	T	A	G	T	T	C	C	C	T	G	G	C	A	A	T	A	A	A	G	G	A	G	A	T	A	T	A	A	A	A	A	A	G	A	A	C	C	C	C
BSNP_IND27	G	C	A	C	C	C	A	C	T	T	T	T	G	T	T	C	T	C	C	T	A	A	C	A	A	T	A	A	A	G	G	A	G	A	T	A	T	A	A	A	A	A	A	G	A	A	C	C	C	C
BSNP_IND28	G	C	A	T	C	C	A	C	T	T	T	T	G	G	T	C	T	C	C	T	G	G	C	A	A	T	A	A	A	G	G	A	G	A	T	A	T	A	A	A	A	A	A	G	A	A	C	C	C	C
BSNP_IND29	G	C	A	C	C	C	A	C	T	T	T	T	G	T	G	C	T	T	C	T	A	A	C	A	A	T	A	G	A	G	A	A	G	A	T	G	C	A	A	A	G	G	T	C	A	A	C	C	T	C
BSNP_IND30	G	C	A	C	C	C	A	C	T	T	T	T	G	T	G	C	T	T	C	T	A	A	C	A	A	T	A	G	A	C	A	A	G	G	T	G	T	A	A	A	A	A	T	G	A	A	C	T	C	G
BSNP_IND31	G	C	G	C	C	C	A	C	T	T	T	C	A	T	G	C	T	T	C	T	A	A	C	A	A	T	A	A	A	C	A	A	G	G	T	A	T	C	A	A	A	A	T	G	A	A	C	T	C	C
BSNP_IND32	G	C	A	T	C	C	A	C	T	T	T	T	A	G	T	T	C	T	C	T	A	A	C	A	A	T	A	G	G	G	G	A	G	A	T	A	C	A	A	A	G	G	T	G	A	G	C	C	T	C
BSNP_IND33	G	C	A	T	C	C	A	C	T	T	T	T	A	G	T	T	C	T	C	T	A	A	C	G	A	T	A	G	G	G	G	G	G	A	T	A	C	A	A	A	G	G	T	G	A	G	C	C	T	C
BSNP_IND34	G	C	A	T	C	C	A	T	T	G	T	T	A	G	T	T	C	T	C	T	A	A	C	A	A	T	A	G	G	G	G	G	G	A	T	A	C	A	A	A	G	G	T	G	A	G	C	C	T	C
BSNP_IND35	G	C	A	T	C	C	A	T	T	G	T	T	A	G	T	T	C	T	C	T	A	A	C	A	A	T	A	G	G	G	G	A	G	G	T	A	C	A	A	A	G	G	T	G	A	G	C	C	T	C
BSNP_IND36	G	C	A	T	C	C	A	C	T	T	T	T	A	G	G	T	C	T	C	T	A	A	C	A	A	T	A	G	G	G	A	G	G	A	T	A	C	A	A	A	A	A	T	G	A	G	C	T	T	C
BSNP_IND37	A	T	A	T	C	C	A	C	T	T	T	C	A	G	T	C	T	T	C	T	A	G	C	A	A	T	G	G	A	G	A	A	G	A	T	A	C	C	A	G	G	A	A	G	A	A	C	T	T	C
BSNP_IND38	G	T	A	T	C	C	A	C	T	T	T	T	A	T	T	C	T	C	C	T	A	G	C	A	A	T	G	G	A	G	A	A	G	A	T	A	C	C	A	A	G	A	A	G	A	A	C	T	T	C
BSNP_IND39	G	T	A	T	C	C	A	C	T	T	C	C	G	G	G	C	T	T	C	T	A	G	C	A	A	T	A	A	A	G	A	A	G	A	T	A	T	A	A	A	G	A	T	G	A	G	C	T	C	C
BSNP_IND40	A	T	A	T	C	C	A	C	T	G	C	T	A	G	T	C	T	C	C	T	G	G	C	A	A	T	A	A	A	G	G	A	G	A	T	A	T	A	A	A	G	A	T	G	A	G	T	C	T	C
BSNP_IND41	G	C	A	C	C	C	A	C	T	G	C	T	G	T	G	T	C	T	C	T	A	A	C	A	A	T	G	G	A	G	A	A	G	A	T	A	T	A	A	G	G	G	T	G	A	A	C	C	T	C
BSNP_IND42	G	C	A	C	C	C	A	C	T	G	C	T	G	T	G	T	C	T	C	T	A	A	C	G	A	T	G	G	A	G	A	A	G	A	T	A	T	A	A	A	G	G	T	G	A	A	C	C	T	C
BSNP_IND43	G	C	A	T	C	C	A	C	T	G	C	T	G	T	T	C	T	T	C	T	G	A	C	A	A	T	A	G	A	C	G	G	G	A	G	G	T	A	A	G	G	G	T	G	A	A	C	C	T	C
BSNP_IND44	G	T	A	T	C	C	A	C	T	G	C	T	G	T	T	C	T	T	C	T	G	A	C	A	A	T	A	G	A	C	G	G	G	A	G	G	T	A	A	G	G	G	T	G	A	A	C	C	T	C
BSNP_IND45	G	C	A	T	C	C	A	C	C	G	T	T	G	T	T	T	C	C	C	T	A	G	C	A	A	T	A	A	A	G	A	A	G	A	T	G	T	C	A	A	A	A	T	G	A	G	T	C	T	C
BSNP_IND46	G	C	A	T	C	C	A	C	C	G	T	T	G	T	T	T	C	C	C	T	A	G	C	A	A	C	A	A	A	G	A	A	G	A	T	G	T	C	A	A	A	A	T	G	A	G	T	C	T	C
BSNP_IND47	G	C	A	T	C	C	A	C	C	G	C	T	G	T	G	C	T	T	C	T	G	G	C	A	A	T	A	G	A	G	G	A	G	G	T	A	T	C	G	A	A	A	T	G	A	G	T	C	T	C
BSNP_IND48	G	C	G	C	C	C	G	C	T	T	T	C	A	G	T	C	T	T	C	T	A	A	C	A	A	T	A	G	A	C	A	A	G	G	T	A	T	A	A	A	A	A	T	G	A	A	C	T	C	C
BSNP_IND49	G	C	G	C	C	C	G	C	T	T	T	C	A	G	T	C	T	T	C	T	A	A	C	A	A	T	A	G	A	C	A	A	G	G	T	A	T	A	A	A	A	A	T	G	A	A	C	T	C	C
BSNP_IND50	G	C	G	C	C	C	G	C	T	T	T	C	A	G	G	C	T	T	C	C	A	A	C	A	A	T	A	G	A	G	A	A	C	G	T	A	T	A	A	G	G	A	T	G	A	A	C	T	C	C
BSNP_IND51	G	C	G	C	T	C	A	C	T	T	T	C	A	G	G	C	T	T	C	T	A	A	C	A	A	T	A	G	A	G	A	A	G	G	T	A	T	A	A	G	G	A	T	G	A	A	C	T	C	C
BSNP_IND52	G	T	A	T	C	C	A	C	C	T	T	C	A	G	T	C	T	T	C	T	A	G	T	A	A	T	G	G	A	G	G	A	C	A	T	A	C	A	A	G	A	A	T	G	A	G	T	C	T	C
BSNP_IND53	G	T	A	T	C	C	A	C	C	T	T	C	A	G	T	C	T	T	C	T	A	G	T	A	A	T	G	G	A	G	G	A	C	A	T	A	C	A	A	A	G	A	T	G	A	A	C	T	T	C
BSNP_IND54	G	T	A	T	C	C	A	C	C	T	T	C	A	G	T	C	T	T	C	T	A	G	T	A	A	T	G	G	A	G	G	A	C	A	T	A	C	A	A	A	A	A	T	G	A	A	C	T	T	C
BSNP_IND55	G	T	A	T	C	C	A	C	C	T	T	C	A	G	T	C	T	T	C	T	A	G	T	A	A	C	G	G	A	G	G	A	C	A	T	A	C	A	A	A	G	A	T	G	A	A	C	T	T	C
BSNP_IND56	G	T	A	T	C	C	A	C	C	T	T	C	A	G	T	C	T	T	C	T	A	G	T	A	A	T	G	G	A	G	G	A	C	A	T	A	C	A	A	A	A	A	T	G	A	G	C	T	T	C
BSNP_IND57	G	T	A	T	C	C	A	C	C	T	T	C	A	T	T	C	T	C	C	T	A	G	T	A	A	T	G	G	A	G	G	A	C	A	T	A	C	A	A	A	G	A	T	G	A	A	C	T	T	C
BSNP_IND58	G	T	A	T	C	C	A	C	C	T	T	C	A	T	T	C	T	C	C	T	A	G	T	A	A	T	G	G	A	G	G	A	C	A	T	A	C	A	A	G	G	A	T	G	A	A	C	T	T	C
BSNP_IND59	G	T	A	T	C	C	A	C	C	T	T	C	A	G	T	C	T	T	C	T	A	G	T	A	A	T	G	G	A	G	G	A	C	A	T	A	C	A	A	A	G	A	T	G	A	A	C	T	T	C
BSNP_IND60	G	T	A	T	C	C	A	C	C	T	T	C	A	G	T	C	T	T	C	T	A	G	C	A	A	T	G	G	A	C	A	A	G	A	T	A	T	A	A	A	G	A	T	G	A	A	C	T	C	C
BSNP_IND61	G	C	G	C	C	C	A	C	T	T	T	T	G	T	T	C	T	T	C	T	A	G	C	A	A	T	A	A	A	C	A	A	G	A	T	G	T	A	A	A	G	A	T	G	A	A	C	T	T	C
BSNP_IND62	G	C	G	C	C	C	A	C	T	T	T	T	G	T	T	C	T	T	C	T	A	G	C	G	A	T	A	A	A	C	A	G	G	A	T	G	T	A	A	A	G	A	T	G	A	A	C	T	T	C
BSNP_IND63	G	C	G	T	C	C	A	C	T	T	T	T	A	G	T	T	C	T	C	T	A	G	T	A	A	T	A	A	A	C	A	A	G	A	T	A	T	A	A	A	A	A	T	G	A	G	C	C	C	C
BSNP_IND64	G	C	G	T	C	C	A	C	T	T	T	T	A	G	T	T	C	T	C	T	A	G	T	A	A	T	G	G	A	G	A	A	C	A	T	A	T	A	A	A	A	A	T	G	A	G	T	C	T	C
BSNP_IND65	G	C	G	T	C	C	A	C	T	T	T	T	A	G	T	T	C	T	C	T	A	G	T	A	A	C	G	G	A	G	A	A	C	A	T	A	T	A	A	A	A	A	T	G	A	G	T	C	T	C
BSNP_IND66	G	C	G	T	C	C	A	C	T	T	T	T	A	G	T	T	C	T	C	T	A	G	T	G	A	T	G	G	A	G	A	A	C	A	T	A	T	A	A	A	A	A	T	G	A	G	T	C	T	C
BSNP_IND67	G	C	G	T	C	C	A	C	T	T	T	T	A	G	T	T	C	T	C	T	A	G	T	A	A	T	G	G	A	G	A	A	C	A	T	A	T	A	A	A	A	A	T	G	A	G	T	C	T	C
BSNP_IND68	G	C	G	T	C	C	A	C	T	T	T	T	A	G	T	T	C	T	C	T	A	G	T	A	A	T	G	G	A	G	A	A	C	A	T	A	T	A	A	A	A	A	T	G	A	G	T	C	T	C
BSNP_IND69	G	C	A	C	C	C	A	C	C	G	C	T	G	G	G	C	T	T	C	C	A	A	C	A	A	T	A	G	A	G	G	A	G	A	T	A	T	A	A	A	G	G	T	G	A	A	C	C	T	C
BSNP_IND70	G	C	A	C	C	C	A	C	C	G	C	T	G	G	G	C	T	T	C	C	A	A	C	A	A	T	A	G	G	G	A	G	G	A	T	A	T	A	A	A	A	G	T	G	A	A	C	C	T	C
BSNP_IND71	G	C	A	C	C	C	A	C	C	G	C	T	A	G	T	C	T	T	C	C	A	A	C	A	A	T	A	G	G	G	A	A	G	A	T	A	T	A	A	A	A	G	T	G	A	A	C	C	T	C
BSNP_IND72	G	C	G	C	C	C	A	C	T	G	C	T	G	G	T	C	C	T	C	T	G	A	C	A	A	T	A	A	A	C	G	A	G	A	T	A	T	A	A	A	G	A	T	G	A	G	C	T	C	C
BSNP_IND73	G	C	A	C	C	C	A	C	C	G	C	T	G	G	G	C	T	T	C	C	A	A	C	A	A	T	A	A	A	C	A	A	C	G	T	G	T	C	A	G	G	A	T	G	A	A	C	T	T	C
BSNP_IND74	G	C	G	C	C	C	A	C	C	G	C	T	G	G	G	C	T	T	C	C	A	A	C	A	A	T	G	A	A	C	A	A	C	A	G	G	T	A	A	G	G	A	T	C	A	A	C	T	T	C
BSNP_IND75	G	C	G	C	T	T	A	T	T	T	T	C	A	G	T	C	T	T	C	T	A	G	C	A	G	T	G	G	A	G	G	A	G	A	T	A	T	A	A	G	A	A	T	G	A	G	T	C	T	C
BSNP_IND76	G	C	G	C	C	C	A	C	T	T	T	C	A	G	T	T	C	C	C	T	A	G	C	A	G	T	A	A	A	C	A	G	G	A	T	A	T	A	A	A	A	A	T	G	A	G	T	C	T	C
BSNP_IND77	G	C	G	C	C	C	A	C	T	T	T	C	A	G	T	T	C	C	C	T	A	G	C	G	G	T	A	A	A	G	A	A	G	A	T	A	T	A	A	A	A	A	T	G	A	G	T	C	T	C
BSNP_IND78	G	C	A	T	C	C	G	C	T	G	C	T	G	T	G	C	T	T	C	C	A	A	C	A	G	T	A	G	A	C	A	A	G	A	T	A	T	A	A	A	A	A	T	G	A	G	C	C	C	C
BSNP_IND79	G	C	A	T	C	C	G	C	T	G	C	T	G	T	G	C	T	T	C	C	A	A	C	G	G	T	A	G	A	C	A	G	G	A	T	A	T	A	A	A	A	A	T	G	A	G	C	C	C	C
BSNP_IND80	G	C	A	T	C	C	G	C	T	G	C	T	G	T	G	C	T	T	C	C	A	A	C	A	G	T	A	G	A	C	A	A	G	A	T	A	T	A	A	A	A	A	T	G	A	G	C	C	C	C
BSNP_IND81	G	T	A	T	C	C	A	C	C	T	T	C	A	T	G	C	T	C	C	C	G	A	C	A	G	T	A	G	A	C	A	A	G	A	T	A	T	A	A	A	A	A	T	G	A	G	C	C	C	C
BSNP_IND82	G	C	G	C	T	C	A	C	T	T	T	C	A	T	G	C	T	C	C	C	A	G	C	A	A	C	A	G	A	G	A	A	G	A	T	A	T	A	A	A	A	A	T	G	G	G	T	C	T	C
BSNP_IND83	G	C	G	T	C	C	A	C	T	T	T	C	A	T	T	C	T	T	C	T	A	A	C	A	G	C	A	G	A	G	G	A	C	A	T	G	T	A	A	A	G	A	T	G	A	G	C	T	C	C
BSNP_IND84	G	C	G	T	C	C	A	C	T	T	T	C	A	T	T	C	T	T	C	T	A	A	C	A	G	C	A	G	A	G	G	A	C	A	T	G	T	A	A	A	G	A	T	G	A	G	C	T	C	C
BSNP_IND85	G	C	G	T	C	C	A	C	T	T	T	C	A	T	T	C	T	T	C	T	A	A	C	A	G	C	A	G	A	G	G	A	C	G	T	G	T	A	A	A	G	A	T	G	A	G	C	T	C	C
BSNP_IND86	G	T	A	T	C	C	A	C	C	T	C	T	A	G	T	C	T	C	C	T	A	G	C	A	A	T	A	G	A	G	G	A	C	A	T	A	T	C	A	G	G	A	T	G	A	A	C	T	T	C
BSNP_IND87	G	T	A	T	C	C	A	C	C	T	C	C	A	G	T	C	T	C	C	T	A	G	C	A	A	T	A	A	A	C	G	A	C	A	T	G	T	C	A	A	G	A	T	G	A	G	T	C	T	C
BSNP_IND88	A	C	A	T	C	C	A	T	T	G	C	T	A	T	T	T	C	T	C	T	A	A	C	G	A	T	A	G	A	G	G	A	C	G	T	A	C	A	A	A	A	A	T	G	A	G	C	T	C	C
BSNP_IND89	G	C	G	T	C	C	A	C	T	G	C	T	A	G	T	T	C	T	A	T	A	A	C	G	A	T	G	G	G	C	A	G	G	A	T	A	T	A	A	G	G	A	T	G	A	A	C	C	C	G
BSNP_IND90	G	C	G	T	C	C	A	C	T	G	C	T	A	G	T	T	C	T	A	T	A	A	C	G	A	T	G	G	G	G	G	A	G	G	T	A	T	A	A	G	G	A	T	G	A	G	C	C	T	C
BSNP_IND91	G	C	G	T	C	C	A	C	T	G	C	T	A	G	T	T	C	T	A	T	A	A	C	G	A	C	G	G	G	G	G	A	G	G	T	A	T	A	A	G	G	A	T	G	A	G	C	C	T	C
BSNP_IND92	G	C	G	T	C	C	A	C	T	G	C	T	A	G	T	T	C	T	A	T	A	A	C	G	A	T	G	G	G	G	G	A	G	G	T	A	T	A	A	G	G	A	T	G	A	G	C	C	T	C
BSNP_IND93	G	T	A	T	C	C	A	C	T	T	C	T	A	G	T	T	C	T	A	T	A	A	C	A	A	T	G	G	A	G	G	A	G	G	T	A	T	C	A	G	G	A	T	G	A	G	C	T	T	C
BSNP_IND94	G	T	G	T	C	C	A	C	T	T	T	T	A	T	T	C	C	T	C	C	A	G	C	G	A	T	A	A	A	G	A	A	G	G	T	A	T	C	A	A	A	A	T	G	A	G	C	C	T	C
BSNP_IND95	A	T	A	T	C	T	A	C	C	G	C	T	G	T	G	T	C	C	C	C	A	A	C	A	A	C	A	G	G	G	A	A	G	A	T	A	T	A	A	A	A	A	T	G	G	G	T	C	T	C
BSNP_IND96	A	T	A	T	C	T	A	C	C	G	C	T	G	T	G	T	C	T	C	T	G	G	C	A	A	T	A	G	A	G	G	A	C	A	T	A	T	A	A	G	A	A	T	G	A	G	T	C	T	C
BSNP_IND97	A	T	A	T	C	T	A	C	C	G	C	T	G	T	G	T	C	T	C	T	G	G	C	A	A	T	A	G	A	G	G	A	C	A	T	A	T	A	A	G	A	A	T	G	A	G	T	C	T	C
BSNP_IND98	G	C	G	C	C	T	A	T	T	T	T	T	A	G	T	C	C	C	C	C	G	A	C	G	A	T	A	G	A	C	A	A	C	A	T	A	T	A	A	A	G	A	A	G	A	G	T	C	T	C
BSNP_IND99	A	C	A	C	T	C	A	C	T	T	T	T	G	G	T	T	C	C	C	T	A	G	C	G	A	C	G	G	A	C	G	A	G	A	T	G	C	C	A	A	G	A	T	G	A	G	C	T	T	C
BSNP_IND100	G	C	A	C	C	C	A	C	T	T	T	T	A	G	T	C	C	T	C	T	G	A	C	A	A	T	A	G	G	G	A	A	G	A	T	G	T	A	A	G	G	A	T	G	A	A	C	T	C	C
BSNP_IND101	G	C	A	T	C	C	A	C	T	T	T	C	A	G	T	C	C	T	C	T	G	A	C	A	A	T	G	G	G	G	A	A	C	A	T	A	T	A	A	A	G	A	T	G	A	G	T	C	T	C
BSNP_IND102	G	C	G	C	C	C	A	C	T	T	T	C	A	G	T	C	T	C	C	T	G	G	C	A	A	T	A	G	A	G	A	A	C	A	T	A	T	A	A	G	G	A	T	G	A	G	C	T	C	C
BSNP_IND103	G	C	G	C	C	C	A	C	T	T	T	C	A	G	T	C	T	C	C	T	G	G	C	A	A	T	A	G	A	G	A	A	G	A	T	A	T	A	A	G	G	A	T	G	A	G	C	T	C	C
BSNP_IND104	G	C	G	C	C	T	A	T	T	T	T	T	G	G	G	C	T	T	C	C	A	A	C	A	A	T	A	G	A	G	A	A	C	G	T	A	T	A	A	G	G	A	T	G	A	A	C	T	C	C
BSNP_IND105	G	C	G	T	C	T	A	C	T	G	C	T	G	T	G	C	T	C	C	T	A	A	C	A	G	C	A	A	A	G	A	A	C	A	T	A	T	A	A	A	G	A	T	G	A	G	C	T	C	C
BSNP_IND106	G	C	G	C	C	T	A	T	T	T	T	T	A	G	T	C	T	C	C	T	A	A	C	A	A	T	A	G	A	C	A	A	G	G	T	G	T	A	A	A	A	A	T	G	A	A	C	T	C	G
BSNP_IND107	G	C	G	C	C	C	A	C	T	T	T	T	G	T	T	C	C	C	C	T	A	A	C	A	A	C	A	G	A	C	A	A	G	G	T	G	T	A	A	A	A	A	T	G	A	A	C	T	C	C
BSNP_IND108	G	C	G	C	C	C	G	C	T	T	T	T	G	T	T	C	T	C	C	T	A	A	C	A	A	C	A	A	A	C	A	G	G	G	T	A	T	A	A	A	A	A	T	G	A	A	C	T	C	C
BSNP_IND109	G	C	G	T	C	C	A	C	T	T	T	T	G	T	G	C	C	C	C	T	A	A	C	G	G	C	A	A	A	C	A	A	G	G	T	A	T	A	A	A	A	A	T	G	A	A	C	T	C	C
BSNP_IND110	G	C	G	T	C	C	A	C	T	T	T	T	G	T	G	C	T	C	C	T	A	A	C	A	A	T	A	A	A	G	A	A	G	G	T	A	T	A	A	A	A	A	T	G	A	G	C	T	T	C
BSNP_IND111	A	T	A	T	C	C	A	C	T	T	T	C	A	G	T	C	T	T	C	T	A	A	C	A	A	T	A	A	A	C	A	A	G	G	T	A	T	A	A	A	A	A	T	G	A	A	C	T	C	C
BSNP_IND112	G	C	A	C	C	C	A	C	T	T	T	C	A	G	T	C	T	C	C	T	A	A	C	A	A	T	A	A	G	G	A	A	G	G	T	A	T	A	A	A	A	A	T	G	A	A	C	C	T	C
BSNP_IND113	G	C	A	C	C	C	A	C	T	T	T	C	A	G	T	C	T	C	C	T	A	A	C	A	A	T	A	A	A	G	A	A	G	G	T	A	T	A	A	A	A	A	T	G	A	A	C	C	T	C
BSNP_IND114	G	C	G	T	C	C	A	C	T	G	C	T	A	T	T	C	T	C	C	C	A	A	C	A	A	T	A	G	G	G	A	A	G	A	T	A	T	C	A	A	A	A	T	G	A	G	C	C	T	C
BSNP_IND115	G	C	G	T	C	C	A	C	T	G	C	T	G	T	G	C	T	C	C	C	A	A	C	A	A	T	A	G	A	G	G	A	G	A	T	A	T	C	A	A	G	A	T	G	A	G	C	C	T	C
BSNP_IND116	G	C	A	T	C	C	A	T	T	G	C	T	A	T	G	C	T	C	C	T	G	G	C	A	A	T	A	G	A	G	A	A	G	A	T	A	T	A	A	G	A	A	T	G	G	G	T	C	T	C
BSNP_IND117	G	C	A	T	C	C	A	T	T	G	C	T	A	T	G	C	T	C	C	T	G	G	C	A	A	T	A	A	A	G	A	A	G	A	T	A	T	A	A	G	A	A	T	G	G	G	T	C	T	C
BSNP_IND118	G	C	A	T	C	C	A	T	T	G	C	T	A	T	G	C	T	C	C	T	G	G	C	A	A	T	A	G	A	G	A	A	G	A	T	A	T	A	A	G	A	A	T	G	G	G	T	C	T	C
BSNP_IND119	G	C	A	C	C	C	A	T	T	G	C	T	A	T	G	C	T	C	C	C	A	A	C	A	A	C	A	A	A	G	A	A	G	A	T	A	T	A	A	A	A	A	T	G	A	G	T	C	T	C
BSNP_IND120	G	C	A	C	C	C	A	T	T	G	C	T	A	T	G	C	T	C	C	C	A	A	C	A	A	T	A	A	A	G	A	A	G	A	T	A	T	A	A	A	A	A	T	G	A	G	T	C	T	C
BSNP_IND121	G	C	A	T	C	C	A	C	T	T	T	T	G	T	G	C	C	C	C	T	A	A	C	A	G	C	A	A	A	C	A	A	C	A	T	A	T	A	A	A	G	A	T	G	A	A	C	T	T	C
BSNP_IND122	G	C	A	T	C	C	A	C	T	T	T	T	G	T	G	C	C	C	C	T	A	A	C	A	G	C	A	A	A	C	A	A	C	A	T	A	T	A	A	A	G	G	T	G	A	A	C	T	T	C
